# Cross-talk-free, high extinction ratio, and ultra-compact all‑optical 4 × 2 encoder using graphene-based plasmonic waveguides

**DOI:** 10.1038/s41598-025-86387-z

**Published:** 2025-01-30

**Authors:** Saima Kanwal, Mohammed R. Saeed, Faris K. AL-Shammri, Mir Hamid Rezaei

**Affiliations:** 1 School of Optical-Electrical and Computer Engineering, University of Shanghai for Science and Technology, Shanghai, 200093 China; 2 Department of Electrical Techniques Engineering, Al-Hussain University College, Kerbala, Iraq; 3 Biomedical Engineering Department, College of Engineering, University of Warith Al Anbiyaa, Karbala, 56001 Iraq; 4 Department of Communications and Electronics, School of Electrical and Computer Engineering, Shiraz University, Shiraz, Iran

**Keywords:** All-optical encoder, High extinction ratio, Plasmonic waveguide, Surface plasmon polariton, Suspended graphene, Cross-talk-free, Optical properties and devices, Integrated optics, Optoelectronic devices and components, Nanophotonics and plasmonics

## Abstract

This paper presents an all-optical 4 × 2 encoder based on graphene-plasmonic waveguides for operation in the wavelength range of 8–12 μm. The basic plasmonic waveguide consists of a silicon (Si) strip and a graphene sheet supported by two dielectric ridges. Surface plasmon polaritons (SPPs) are stimulated in the spatial gap between the graphene sheet and the Si strip. The effect of geometric parameters and chemical potential of the graphene sheet changes on the suggested waveguide’s waveguiding behavior is meticulously investigated using the three-dimensional finite-difference time-domain (3D-FDTD) method. The encoder comprises a straight waveguide to detect the state of the In_0_ input and two Y-combiners with outputs Out_0_ and Out_1_ to detect the state of the In_1_, In_2_, and In_3_ inputs. The encoder exhibits a minimum extinction ratio (*ER*_*min*_) of 19 dB at a wavelength of 10 μm. In addition, the cross-talk (*CT*) and insertion loss (*IL*) values are −21.3 and −1.31 dB, respectively. The encoder offers an ultra-compact structure with a total footprint of 4.25 μm^2^. Due to its exceptional waveguiding features, low *CT* and *IL* values, and high *ER*_*min*_, the proposed encoder holds promise for various communication and signal processing applications.

## Introduction

Plasmonic devices are specialized structures capable of trapping light within subwavelength regions, significantly reducing the size of the overall structure^1–3^. These devices are adept at generating surface plasmon polaritons (SPPs) at the interface between metal and dielectric materials, facilitating their propagation along this boundary. However, within the materials themselves, the electromagnetic field attenuates severely^4–6^. The term “polariton” denotes the oscillation of the metal’s bound electrons in conjunction with the excitation of photons. In this scenario, photons are responsible for the excitation of surface plasmons, leading to the use of “plasmon polariton” to describe the interaction between a photon and a plasmon^7^. Noble metals such as gold (Au) and silver (Ag) are typically employed as plasmonic materials in the visible and near-infrared (NIR) spectrum to generate SPPs. However, these materials are not suitable for mid-infrared (MIR) to terahertz (THz) frequencies due to their high losses^8–10^. The introduction of graphene has revolutionized the fields of electronics and photonics. Graphene is distinguished by its remarkable electrical and optical characteristics, paving the way for its incorporation into various optoelectronic devices^10,11^. It is characterized by its ability to confine light in areas smaller than the stimulating wavelength, minimal losses, and a refractive index that can be adjusted through chemical doping or external voltage^12,13^. Graphene’s unique blend of optical and electrical properties renders it an exemplary material for optoelectronic applications. Plasmonic waveguides can be generally classified into four main categories: metal-insulator (MIM)^14^, insulator-metal-insulator (IMI)^15^, hybrid plasmonic^16^, and graphene-based plasmonic waveguides^17^, each category being suited for special wavelengths and applications.

Encoders are one of the most well-known digital devices in telecommunications and signal processing systems. An encoder has 2^*n*^ inputs and *n* outputs, and only one of the inputs can be in the logical state “1” at any time. Optical encoders have been reported based on various structures, such as photonic crystals (PhCs)^18,19^, plasmonics^20,21^, and other schemes^22–24^. Moniem proposed a 4 × 2 optical encoder based on two-dimensional PhCs with a switching speed of 500 GHz and a total footprint of 1225 *μ*m^2^^25^. Abdulwahid et al. reported a 4 × 2 optical encoder using hybrid plasmonic waveguides^26^. It operates at the wavelength of 1310 nm with a maximum transmission value of 68.4%, a transmission threshold of 30%, and a footprint of 0.368 *μ*m^2^. However, it requires two extra input control signals to function correctly. Haddadan and Soroosh designed a plasmonic priority encoder using graphene nanoribbons^27^. The encoder exhibits a contrast ratio of 13.1 dB and a cross-talk of −16.22 dB with a whole area of 1.92 *μ*m^2^, operating at a wavelength of 7 *μ*m. However, this encoder requires six graphene nanoribbons with different chemical potentials. As a result, the lack of designing an encoder with acceptable characteristics, minimum input signals, and a straightforward fabrication process is felt. In this paper, we design a 4 × 2 optical encoder based on suspended graphene plasmonic waveguides operating in the wavelength range of 8 to 12 *μ*m. The performance of the proposed encoder is analyzed using the three-dimensional finite-difference time-domain (3D-FDTD) method.

The rest of the paper is structured as follows: The basic suspended graphene plasmonic waveguide is presented in Section “The basic suspended graphene plasmonic waveguide”. In this section, the effects of the geometrical parameters and chemical potential of graphene on the properties of the waveguide are investigated. The encoder structure and simulation results obtained by the 3D FDTD method are presented in Section “Proposed 4×2 plasmonic encoder”. Finally, the conclusion is expressed in Section “Conclusion”.

## The basic suspended graphene plasmonic waveguide

**Fig. 1:**
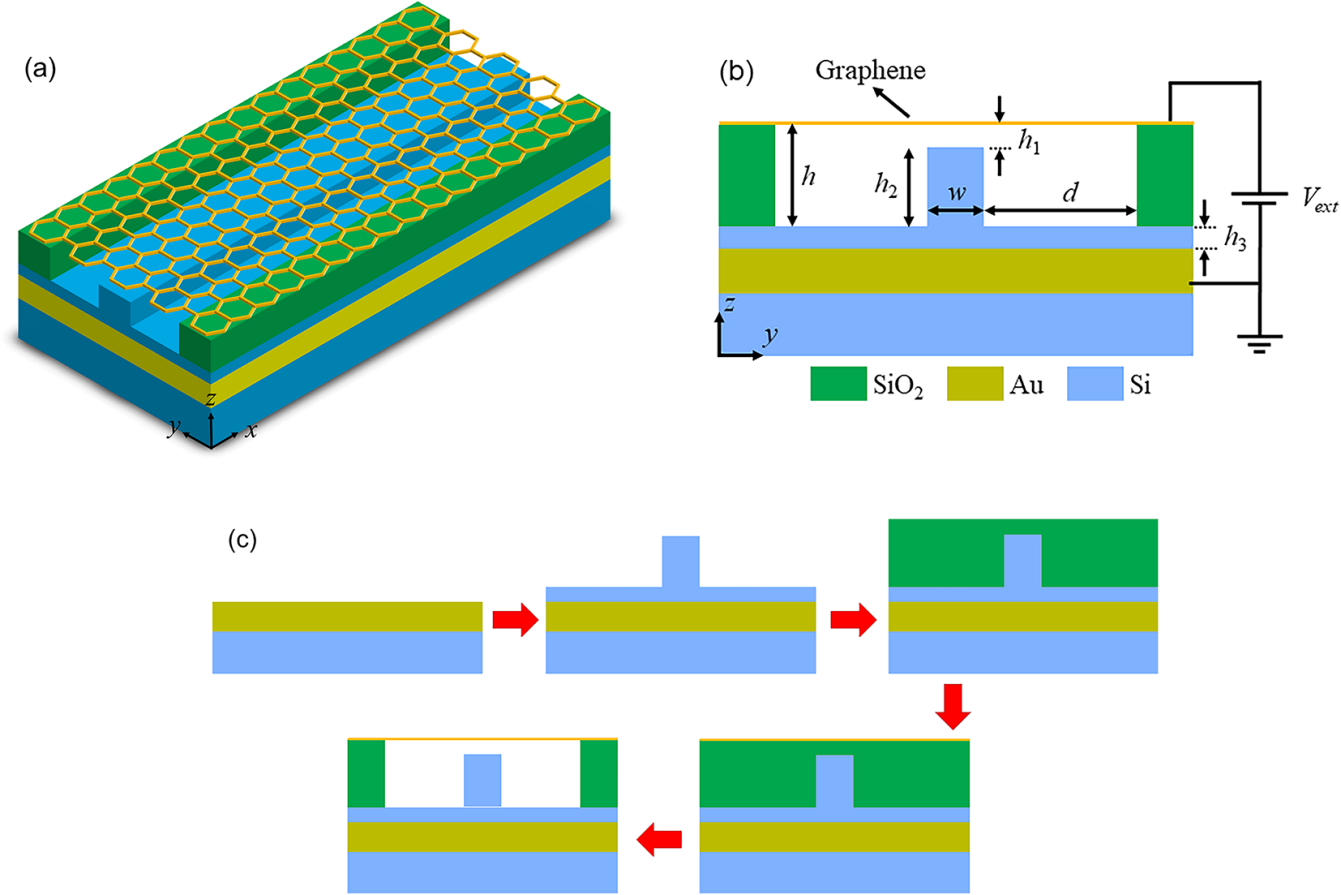
(**a**) 3D schematic of the basic suspended graphene plasmonic waveguide. (**b**) The cross-sectional view of the waveguide with the geometric parameters. (**c**) The possible steps of the fabrication process of the basic waveguide.

Figure 1a shows schematically the proposed basic plasmonic waveguide. It consists of an Au layer on a dielectric substrate, a thin silicon (Si) spacer layer, a Si strip at the center of the waveguide, and a graphene sheet supported by two dielectric ridges. As seen in Fig. 1b, the geometric parameters are the gap between the graphene sheet and the Si spacer layer (*h*), which includes a gap between the graphene sheet and the Si strip, and the height of the Si strip (*h*_1_), the width of the Si strip (*h*_2_), the height of the Si spacer layer (*h*_3_), and the distance of the dielectric ridges to the Si strip (d). The parameter d is chosen large enough that the existence of the dielectric ridges does not affect the propagation mode of the waveguide. In Fig. 1c, the possible fabrication processes of the suggested waveguide are plotted. The steps can be as follows: (i) deposition of the Au layer on the dielectric substrate via thermal atomic layer deposition and sputtering methods^28,29^, (ii) deposition of Si and patterning it to for the spacer layer and the strip using e-beam lithography^30,31^, (iii) deposition of the SiO_2_ layer using plasma-enhanced chemical vapor deposition (PECVD)^32,33^, (iv) transfer of the CVD graphene grown on copper onto the SiO_2_ layer by employing the wet transfer method^34^, and (v) selective etching of the middle part of the SiO_2_ layer by dipping the entire device into 1:6 buffered oxide etch (BOE), which uniformly removes SiO_2_ across the substrate, including the area below the graphene sheet^35^. The fabrication process of the proposed suspended graphene-based waveguide and its experimental results have been detailed in Ref.^35^.

The other affecting parameter is the chemical potential (*μ*_*c*_) of the graphene sheet. Applying chemical doping and an electrostatic field varies the chemical potential of graphene by changing the number of charge carriers, as follows^36^:

1 $$n = \frac{2}{{\pi \hbar^{2} \nu_{F}^{2} }}\int_{0}^{\infty } {E\left[ {f\left( E \right) - f\left( {E + 2\mu_{c} } \right)} \right]} dE$$

where *ħ* is the reduced Planck’s constant, *v*_*F*_ = 10^6^ m/s is the Fermi velocity, and $f\left( E \right) = \left\{ {1 + \exp \left[ {\left( {E - \mu_{c} } \right)/k_{B} T} \right]} \right\}^{ - 1}$ is the Fermi distribution function, *k*_*B*_ is the Boltzmann’s constant, and *T* is temperature. The complex permittivity of graphene (*ε*_*g*_) is expressed in terms of the background relative permittivity (*ε*_*r*_), surface conductivity of graphene (*σ*_*g*_), free space (*ε*_0_) permittivity, operation angular frequency (*ω*), and graphene thickness (Δ) as follows^37^:

2 $$\varepsilon_{g} = \varepsilon_{r} + i\frac{{\sigma_{g} }}{{\varepsilon_{0} \omega \Delta }}$$

The surface conductivity of graphene defined by the Kubo formula consists of two parts, inter-band (*σ*_g-inter_) and intra-band (*σ*_g-intra_), calculated by the following equations^17^:

3 $$\sigma_{{g{\text{ - inter}}}} = \frac{{ie^{2} }}{4\pi \hbar }\ln \left( {\frac{{2\left| {\mu_{c} } \right| - \left( {\omega + i\tau^{ - 1} } \right)\hbar }}{{2\left| {\mu_{c} } \right| + \left( {\omega + i\tau^{ - 1} } \right)\hbar }}} \right)$$

4 $$\sigma_{{g - {\text{intra}}}} = \frac{{ie^{2} k_{B} T}}{{\pi \hbar^{2} \left( {\omega + i\tau^{ - 1} } \right)}}\left[ {\frac{{\mu_{c} }}{{k_{B} T}} + 2\ln \left( {1 + e^{{ - \frac{{\mu_{c} }}{{k_{B} T}}}} } \right)} \right]$$

Here, *e* is the electron charge, and *τ* donates the relaxation time, which in the wavelength range of MIR to THz is expressed by^38^:

5 $$\tau = \, \left( {2\Gamma } \right)^{ - 1} = \frac{{\mu_{m} \mu_{c} }}{{e\nu_{F}^{2} }}$$

where Г and *μ*_*m*_ are the scattering rate and graphene mobility, respectively. The mobility of carriers in graphene can be changed from 1000 cm^2^/V.s when manufactured through CVD to 230000 cm^2^/V.s for suspended and exfoliated graphene^35^. As mentioned, altering the carrier density in graphene through chemical doping or applying an external voltage can also modify its chemical potential, affecting its conductivity. The relationship between the chemical potential of graphene and the external voltage (*V*_*ext*_) is as follows^39,40^:

6 $$\mu_{c} = \hbar v_{F} \sqrt {\pi \left( {n_{0} + \frac{{C_{p} \left| {V_{ext} } \right|}}{e}} \right)}$$

where *n*_0_ is the intrinsic carrier concentration of graphene and *C*_*p*_ is the capacitance in F/m^2^, corresponding to the capacitor formed by the suspended graphene sheet, the Au metal, and the dielectrics between them. As demonstrated in Fig. 1b, the alteration of external voltage results in a corresponding change in the charge stored within the capacitor. This fluctuation in charge carriers directly influences the chemical potential of graphene, subsequently modifying its surface conductivity. Consequently, this leads to a variation in the permittivity of graphene, which in turn affects the effective refractive index and the overall optical characteristics of the structure. Thus, it can be deduced that the performance of the device can be effectively controlled through the application of an external voltage between the graphene sheet and the metal layer.

In our simulations, we model Si and Au materials using the Palik^41^ and CRC^42^ models, respectively. To simulate the waveguide, we used the commercial software Ansys Lumerical and the 3D-FDTD method^43^. This software offers a comprehensive set of tools for simulating complex photonic devices. Additionally, we set perfectly matched layers in all directions as boundary conditions to prevent light reflection towards the encoder. We also utilized a local fine mesh surrounding the graphene sheet to achieve accurate results.

**Fig. 2:**
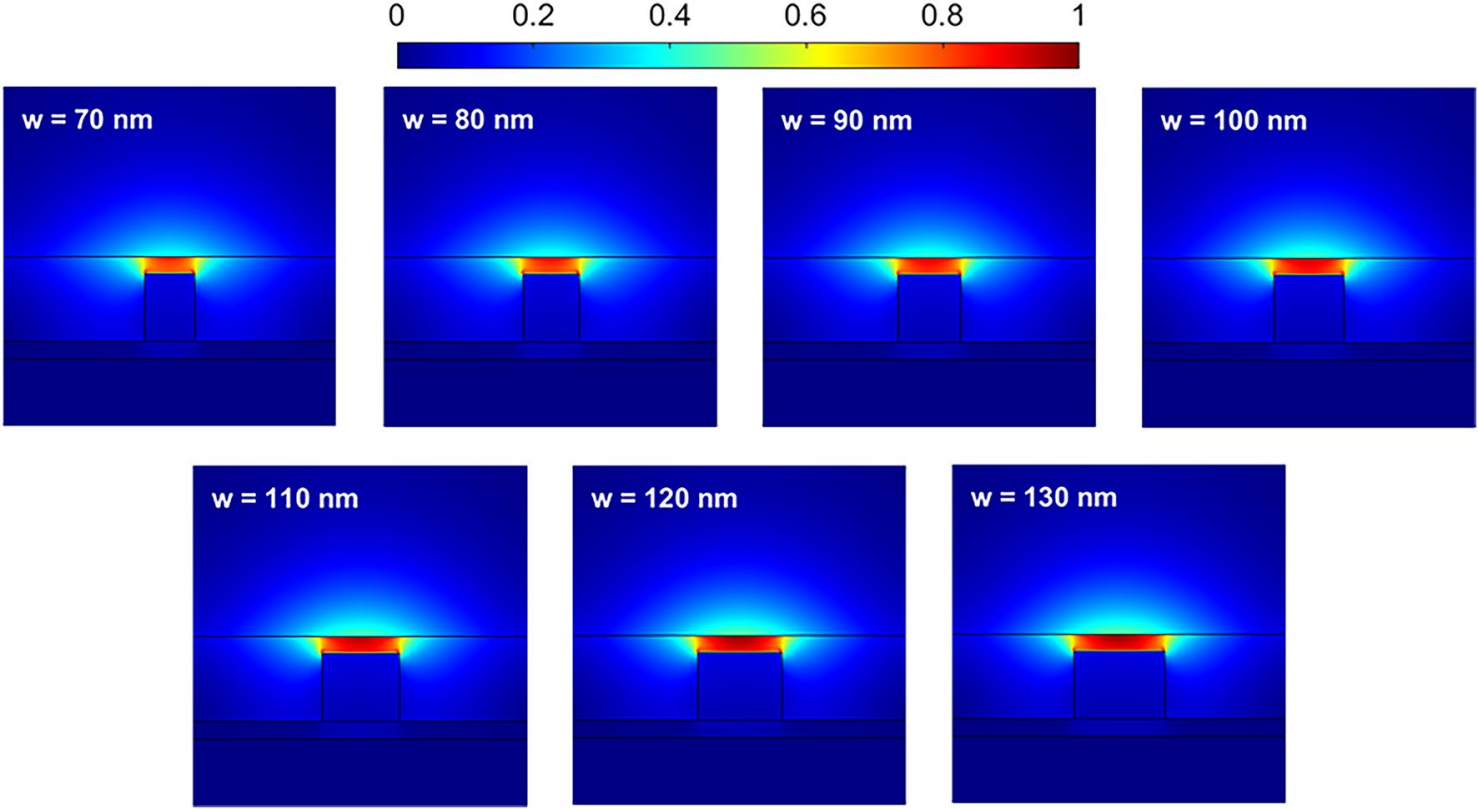
Distribution of the electric field |*E*| for different values of the *w* parameter. The other parameters are *h* = 100 nm, *h*_1_ = 20 nm, *h*_3_ = 20 nm, *μ*_*c*_ = 0.6 eV, and λ = 10 *μ*m.

In the following, the effects of geometric parameters on the features of the basic waveguide are studied. Figure 2 shows the distribution of the magnitude of the electric field |*E*| for the fundamental transverse magnetic (TM) mode as a function of the Si strip width in the *z*-*y* plane at λ = 10 *μ*m. The width changes from 70 to 130 nm with steps of 10 nm. The parameters *h*, *h*_1_, *h*_2_, *h*_3_, and *μ*_*c*_ are 100, 20, 80, 20 nm, and 0.6 eV, respectively. The field intensity increases at the center of the stimulated mode as the width increases. Additionally, the waveguide acts as a single-mode waveguide with a width of 120 nm in the wavelength range of 8 *μ*m to 12 *μ*m. For *w* = 130 nm, the second-order mode is also excited at λ = 8 *μ*m. Therefore, the waveguide is single-mode for *w* = 120 in the wavelength range of 8–12 μm.

**Fig. 3:**
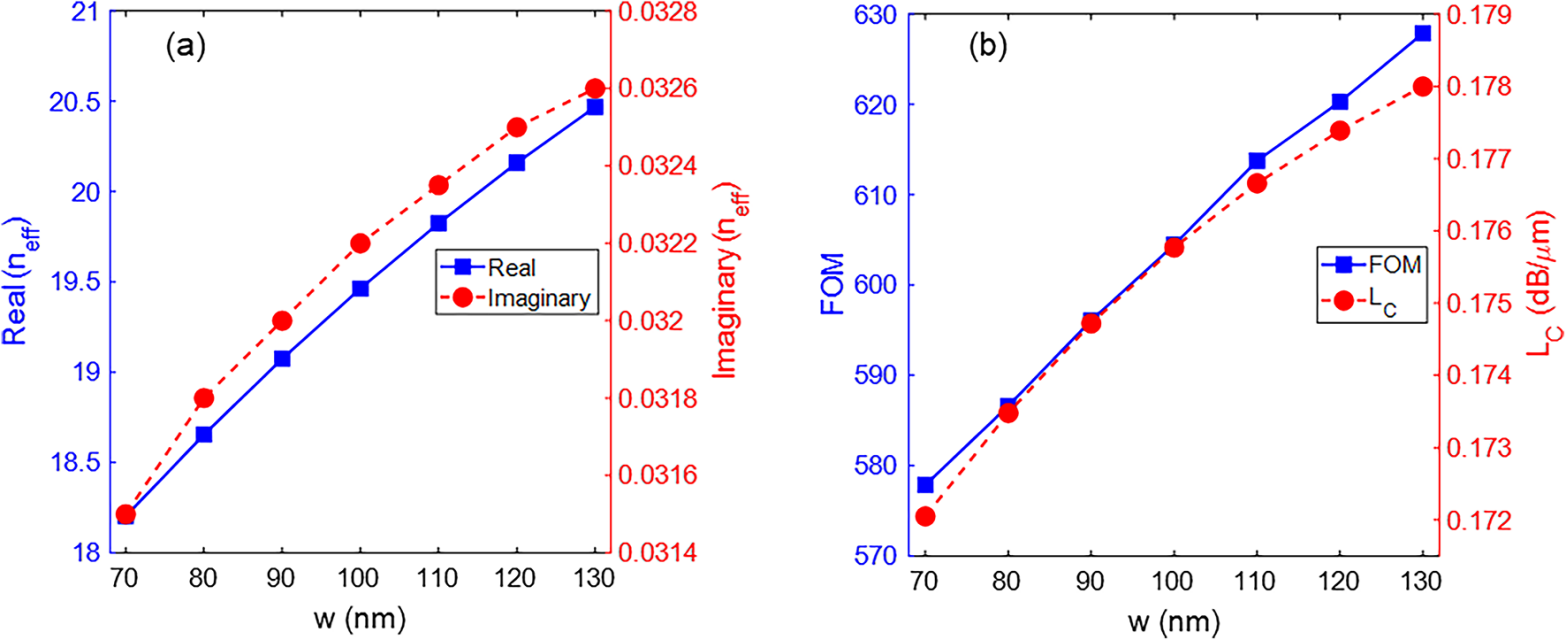
(**a**) Real and imaginary parts of the *n*_*eff*_ and (**b**) the FOM and *L*_*C*_ of the waveguide as a function of the *w* parameter. The other parameters are *h* = 100 nm, *h*_1_ = 20 nm, *h*_3_ = 20 nm, *μ*_*c*_ = 0.6 eV, and λ = 10 *μ*m.

The graphs in Fig. 3a demonstrate the real and imaginary parts of the effective refractive index (*n*_*eff*_) of the fundamental mode for *w* variations. The real part is more sensitive to changes in the *w* parameter than the imaginary part. Using linear approximation, the rate of change of the real part to *w* is *d*[Re(*n*_*eff*_)]/*dw* = 3.776 × 10^−2^ nm^−1^. Conversely, the imaginary part has an almost constant value of Im(*n*_*eff*_) ≈ 0.032. To have criteria for evaluating the performance of the proposed waveguide, two parameters called figure-of-merit (FOM)^44^ and confinement loss (*L*_*C*_)^45^ are defined as follows:

7 $$FOM = \frac{{{\text{Re}} \left( {n_{eff} } \right)}}{{{\text{Im}} \left( {n_{eff} } \right)}}$$

8 $$L_{C} {\text{(dB/}}\mu {\text{m)}} = \frac{20}{{\ln \left( {10} \right)}}\frac{2\pi }{\lambda }{\text{Im}} \left( {n_{eff} } \right) \,$$

where λ is given in *μ*m. The FOM represents the propagation length normalized to the SPP’s wavelength, and the *L*_*C*_ represents the losses arising from the leaky nature of the modes and the non-perfect structure of the waveguides. It is obvious that higher values of FOM and lower values of *L*_*C*_ are desirable. Figure 3b shows the FOM and *L*_*C*_ of the waveguide as a function of *w* variations. The highest value of FOM = 627.9, achieved for *w* = 130 nm at λ = 10 *μ*m. However, the waveguide becomes multimode for *w* = 130 nm at λ = 8 *μ*m. The FOM for *w* = 120 nm is 620.3 and does not differ significantly from that for *w* = 130 nm. In addition, the *L*_*C*_ has a negligible change for *w* variations due to the almost constant value of the Im(*n*_*eff*_).

**Fig. 4:**
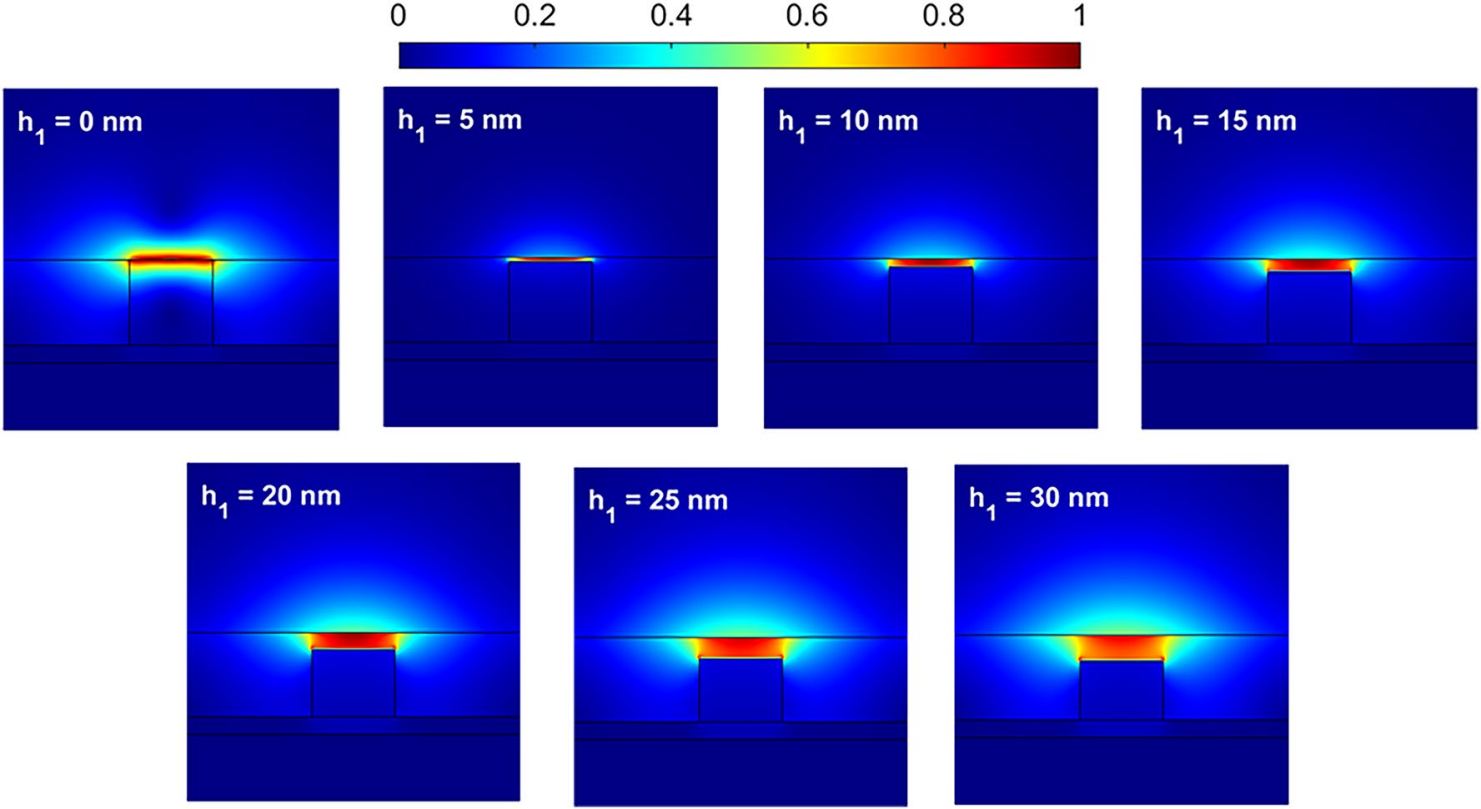
Distribution of the electric field |*E*| for different values of the *h*_1_ parameter at λ = 10 *μ*m. The other parameters are *w* = 120 nm, *h* = 100 nm, *h*_3_ = 20 nm, and *μ*_*c*_ = 0.6 eV.

**Fig. 5:**
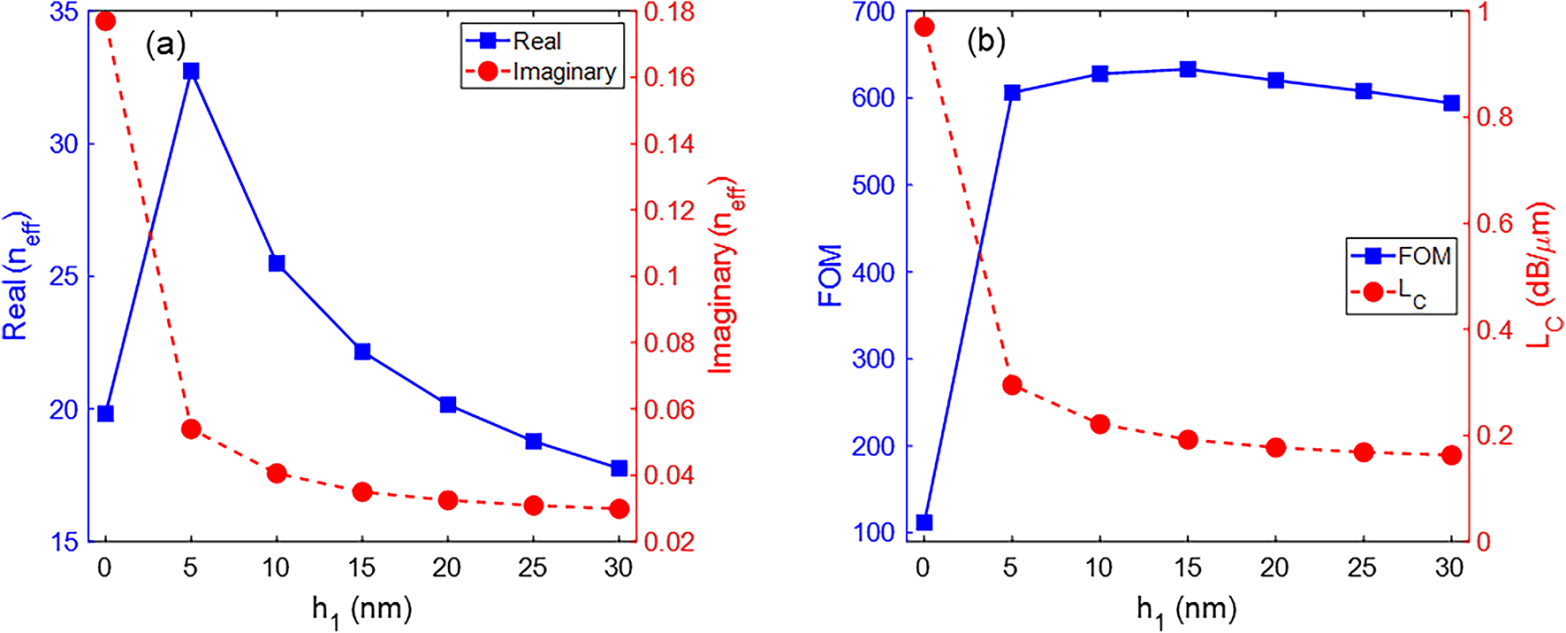
(**a**) Real and imaginary parts of the *n*_*eff*_ and (**b**) the FOM and *L*_*C*_ of the waveguide as a function of the *h*_1_ parameter. The other parameters are *w* = 120 nm, *h* = 100 nm, *h*_3_ = 20 nm, *μ*_*c*_ = 0.6 eV, and λ = 10 *μ*m.

Figure 4 illustrates the distribution of |*E*| as a function of the gap between the graphene sheet and the Si strip. The waveguide parameters are *w* = 120 nm, *h* = 100 nm, *h*_3_ = 20 nm, *μ*_*c*_ = 0.6 eV, and λ = 10 *μ*m. The gap (*h*_1_) increases from 0 to 30 nm with steps of 5 nm. For the case of *h*_1_ = 0, the graphene sheet is placed on the Si strip, and there is no gap. Therefore, the permittivity of the graphene sublayer changes from that of air to that of Si. It causes a significant change in the electric field distribution of the fundamental mode. The electric field intensity is maximum in the graphene sheet and gradually decreases in the surrounding ambient. When *h*_1_ increases, the electric field is strongly confined to the gap area. The higher the value of *h*_1_, the less the confinement of mode and the lower the intensity of the field at the center of the waveguide. Figure 5a shows the real and imaginary parts of the *n*_*eff*_ for different values of *h*_1_ from 0 to 30 nm. A comparison between curves in Figs. 3a, 5a reveals that changes in *h*_1_ have a more significant effect on the real and imaginary parts of the *n*_*eff*_ than changes in *w*. When *h*_1_ = 0, the waveguide has the highest value of the imaginary part, which indicates higher waveguide losses. Creating a gap between the graphene sheet and the Si strip changes the boundary conditions for exciting SPP waves, leading to a significant difference in the real value of *n*_*eff*_. As *h*_1_ increases, the cross-sectional area of the stimulated mode also increases, causing a reduction in light confinement and resulting in a decrease in the real part of *n*_*eff*_. Additionally, the electric field spreads across more space in the ambient region of the structure as *h*_1_ increases. In Fig. 5b, the FOM and *L*_*C*_ of the waveguide as a function of *h*_1_ is displayed. Except for *h*_1_ = 0, the FOM varies around 600 for other values of *h*_1_. Also, the *L*_*C*_ decreases from 0.97 dB/*μ*m to 0.163 dB/*μ*m when *h*_1_ increases from 0 to 30 nm. Generally, there is no significant difference for *h*_1_ changes from 5 to 30 nm.

**Fig. 6:**
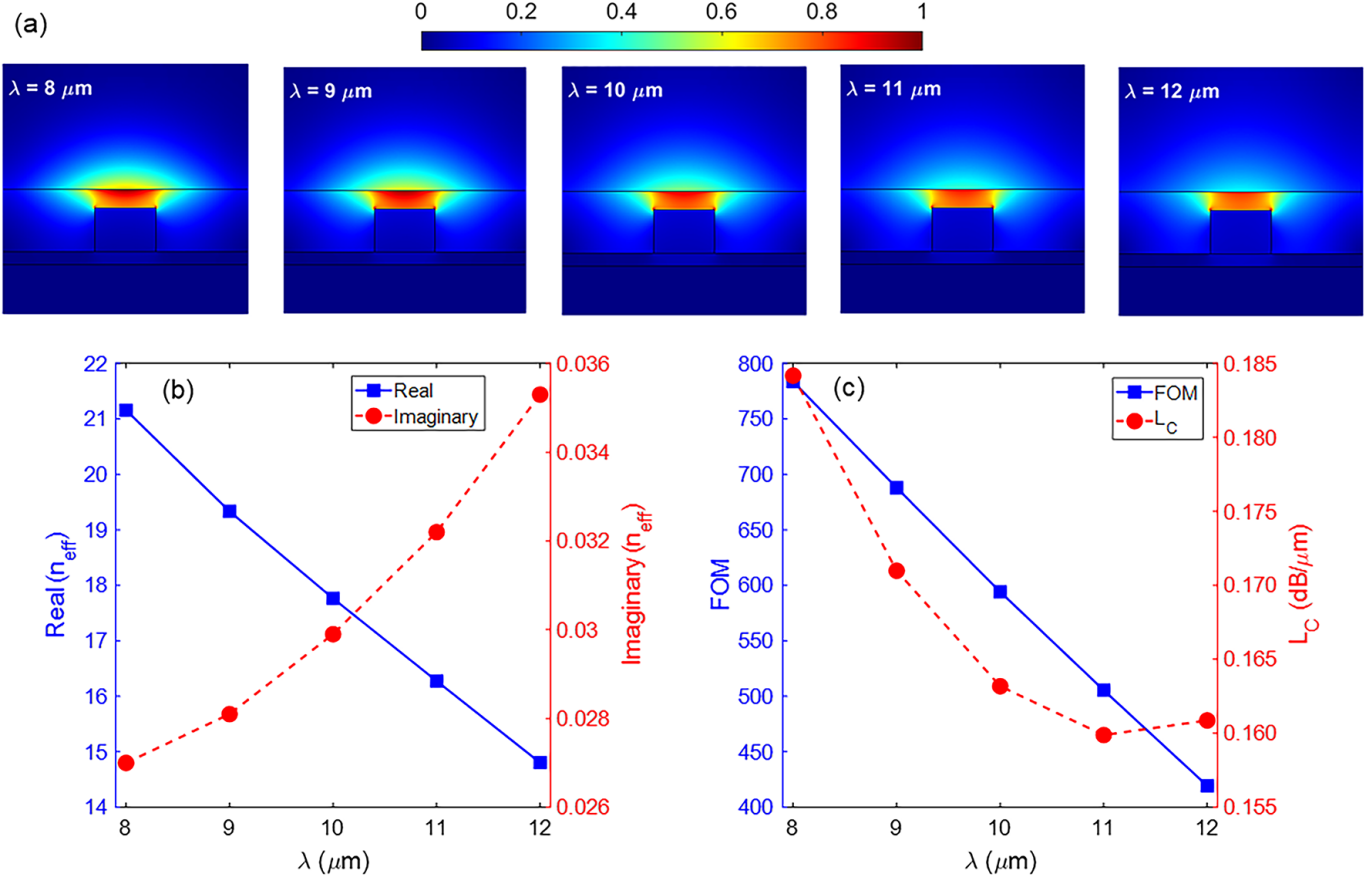
(**a**) Distribution of the electric field |*E*| at different wavelengths, (**b**) the real and imaginary parts of the *n*_*eff*_, and (**c**) the FOM and *L*_*C*_ parameters of the waveguide as a function of wavelength. The other parameters are *w* = 120 nm, *h* = 100 nm, *h*_1_ = 30 nm, *h*_3_ = 20 nm, and *μ*_*c*_ = 0.6 eV.

In Fig. 6a, the distribution of |*E*| for the fundamental TM mode is depicted at five wavelengths. Additionally, Fig. 6b illustrates the real and imaginary parts of the *n*_*eff*_ of the fundamental mode. The real part exhibits a decrease with a rate of −1.588 *μ*m^−1^, as evident from the electric field intensity at the center of the waveguide in (Fig. 6a). Meanwhile, the imaginary part of *n*_*eff*_ shows a negligible change as the working wavelength varies. Figure 6c illustrates the FOM and *L*_*C*_ of the basic waveguide as a function of the operation wavelength. It is worth noting that the Im(*n*_*eff*_) also increases with increasing wavelength. However, this does not mean a linear upward trend in the *L*_*C*_ because the *L*_*C*_ parameter depends on the wavelength. For this reason, the highest and lowest values of the *L*_*C*_ are equal to 0.1842 and 0.1599 dB/*μ*m, observed at λ = 8 *μ*m and λ = 11 *μ*m, respectively.

**Fig. 7:**
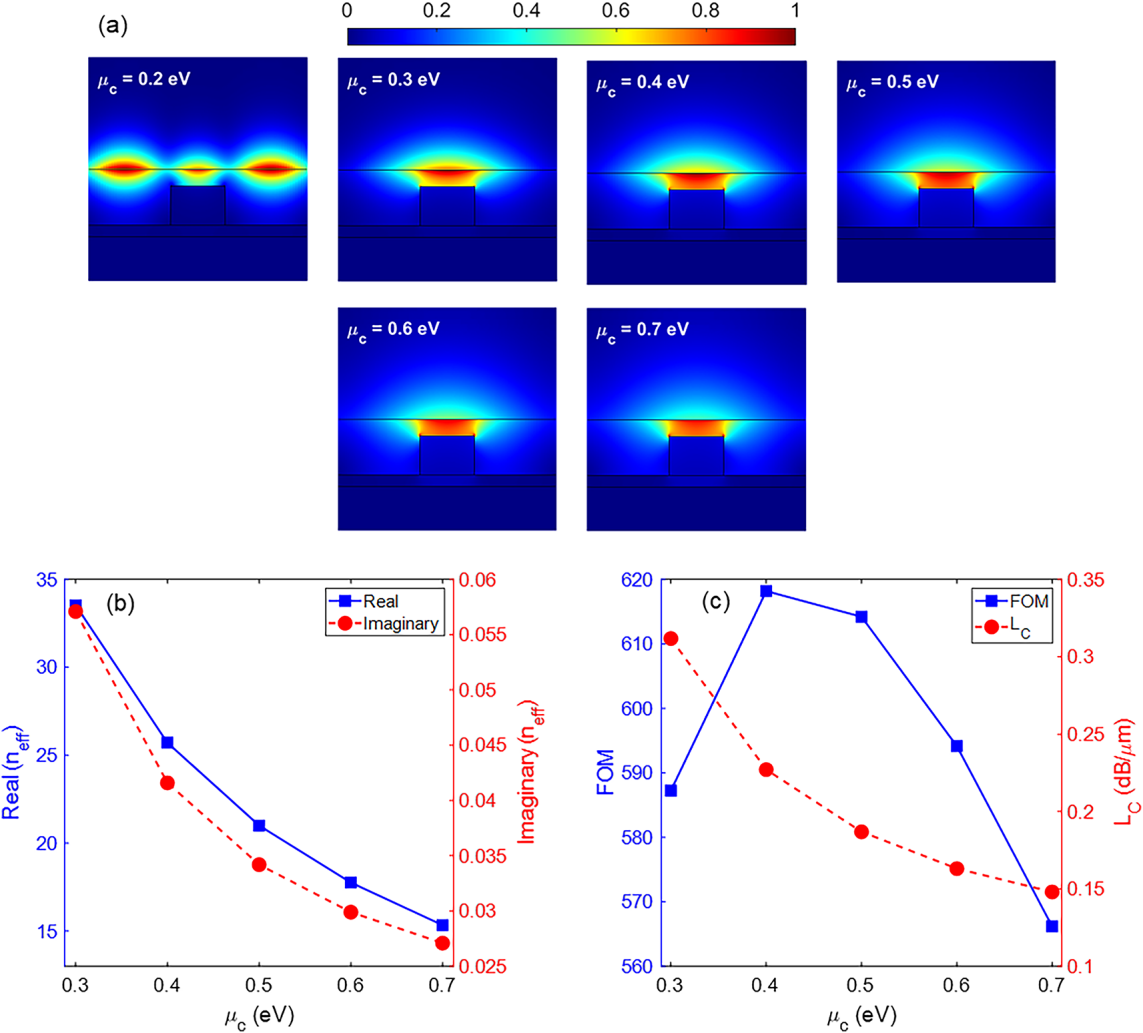
(**a**) Distribution of the electric field |*E*| for different values of the *μ*_*c*_ parameter, (**b**) the real and imaginary parts of the *n*_*eff*_, and (**c**) the FOM and *L*_*C*_ parameters of the waveguide as a function of the *μ*_*c*_ parameter. The other parameters are *w* = 120 nm, *h* = 100 nm, *h*_1_ = 30 nm, *h*_3_ = 20 nm, and λ = 10 *μ*m.

In addition to the geometric variables, the chemical potential of the graphene sheet is also effective in its waveguiding behavior. Changing the chemical potential of graphene causes a change in its surface conductivity and, consequently, the permittivity of graphene. In Fig. 7a, the distributions of |*E*| for different values of the chemical potential of graphene from 0.2 to 0.7 eV at λ = 10 *μ*m are observed. Although the waveguide acts as a multimode waveguide for *μ*_*c*_ = 0.2 eV, it is a single-mode waveguide for higher values of *μ*_*c*_. Figure 7b presents the real and imaginary parts of the *n*_*eff*_ as a function of *μ*_*c*_. The higher the *μ*_*c*_ value, the lower the real and imaginary parts of the *n*_*eff*_. However, FOM gives a better criterion to choose a proper value of *μ*_*c*_. The highest FOM is 618.2, which is obtained for *μ*_*c*_ = 0.4 eV (Fig. 7c).

## Proposed 4 × 2 plasmonic encoder

**Fig. 8:**
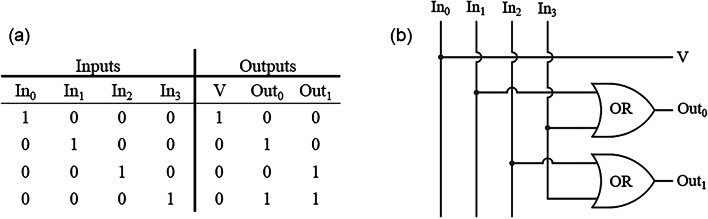
(**a**) Modified truth table and (**b**) the block diagram of a 4 × 2 encoder with an extra output.

A 4 × 2 encoder has four inputs and two outputs. At any time, only one of the inputs is ON, and the outputs generate the binary code corresponding to the inputs. It is worth noting that the terms “ON” and “OFF” refer to logical “1” and “0” states, respectively. When the In_0_ input is ON, the outputs Out_0_ and Out_1_ are OFF. Therefore, to distinguish whether the input In_0_ is OFF or ON, an extra output called V is added to the structure. If the V output is ON, the In_0_ input is ON; otherwise, the In_0_ input is OFF. The modified truth table of a 4 × 2 encoder and its corresponding block diagram are provided in (Fig. 8).

**Fig. 9:**
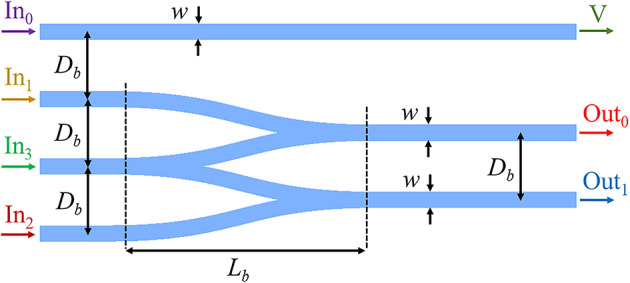
Schematic of the Si Strips of the encoder, consisting of a straight waveguide and two stick-together Y-combiners. The other layers, including the Au and spacer layers, as well as the graphene sheet, have not been plotted for better visibility.

The schematic of the Si strips of the proposed encoder is shown in (Fig. 9). It consists of four input ports named In_0_, In_1_, In_2_, and In_3_ and three output ports named V, Out_1_, and Out_2_. Two stick-together bent Y-combiners are used to implement OR logic gates, and a straight waveguide is used to detect the In_0_ input. The distance between the centers of the input ports and the length of the bent section of the Y-combiners are denoted by *D*_*b*_ and *L*_*b*_, respectively. The layer arrangement and other structural parameters of the encoder are the same as those of the basic waveguide: *w* = 120 nm, *h*_1_ = 5 nm, *h*_2_ = 95 nm, *h*_3_ = 20 nm, and *μ*_*c*_ = 0.4 eV. It should be noted that two bent Y-combiners are used in the proposed encoder structure. In curved structures, the possibility of converting the guided mode into a radiation one increases with the reduction of light confinement. Although the highest FOM is attained at *h*_1_ = 15 nm, the optimal real part of neff shown in Fig. 5a corresponds to *h*_1_ = 5 nm. Therefore, this value has been selected for the h1 parameter. The initial values of *D*_*b*_ and *L*_*b*_ are selected as 500 nm and 2 *μ*m, respectively.

**Fig. 10:**
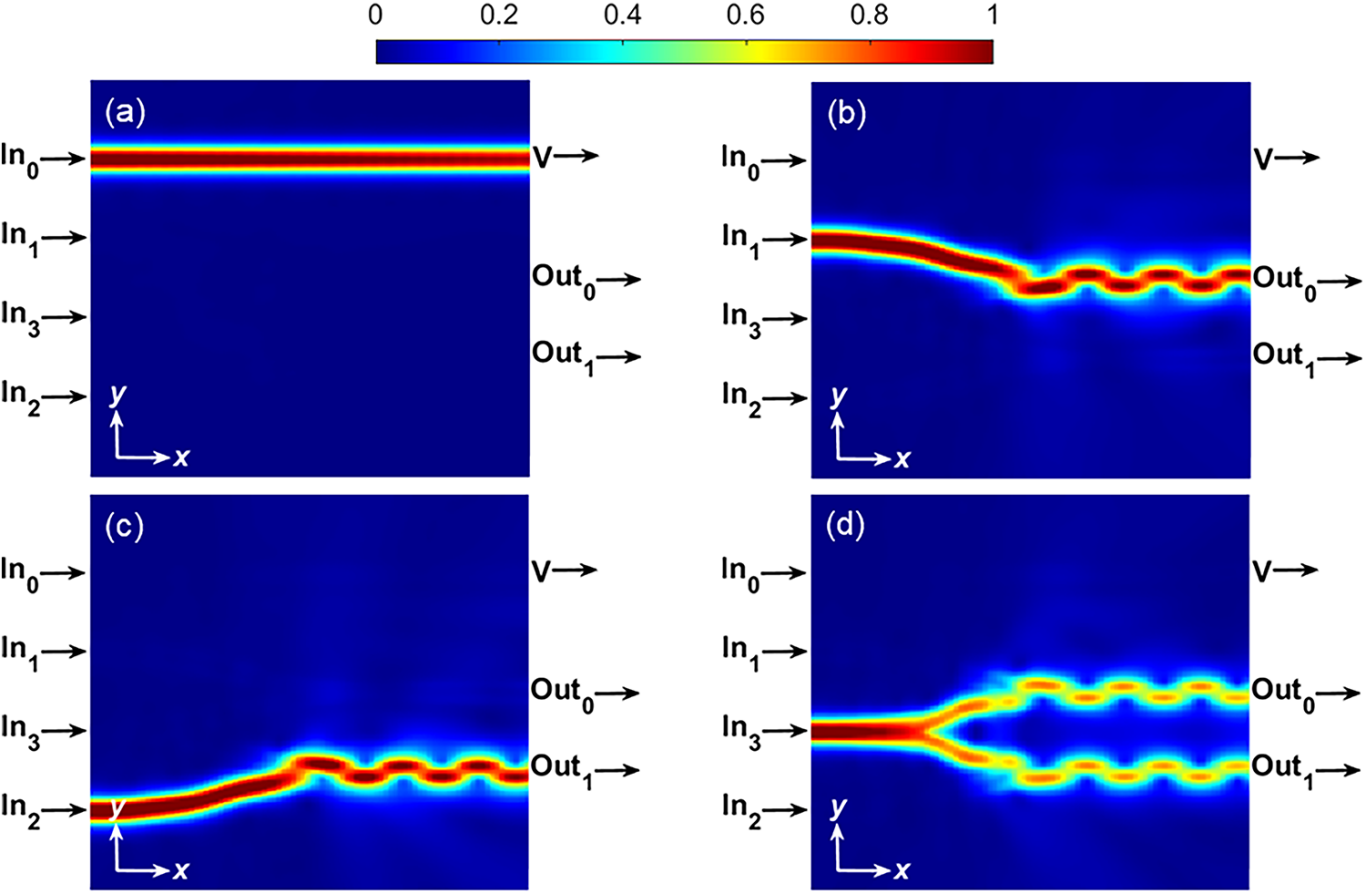
Distribution of the electric field |*E*| for different conditions of the proposed encoder (**a**) In_0_ = ON, (**b**) In_1_ = ON, (**c**) In_2_ = ON, and (**d**) In_3_ = ON.

**Fig. 11:**
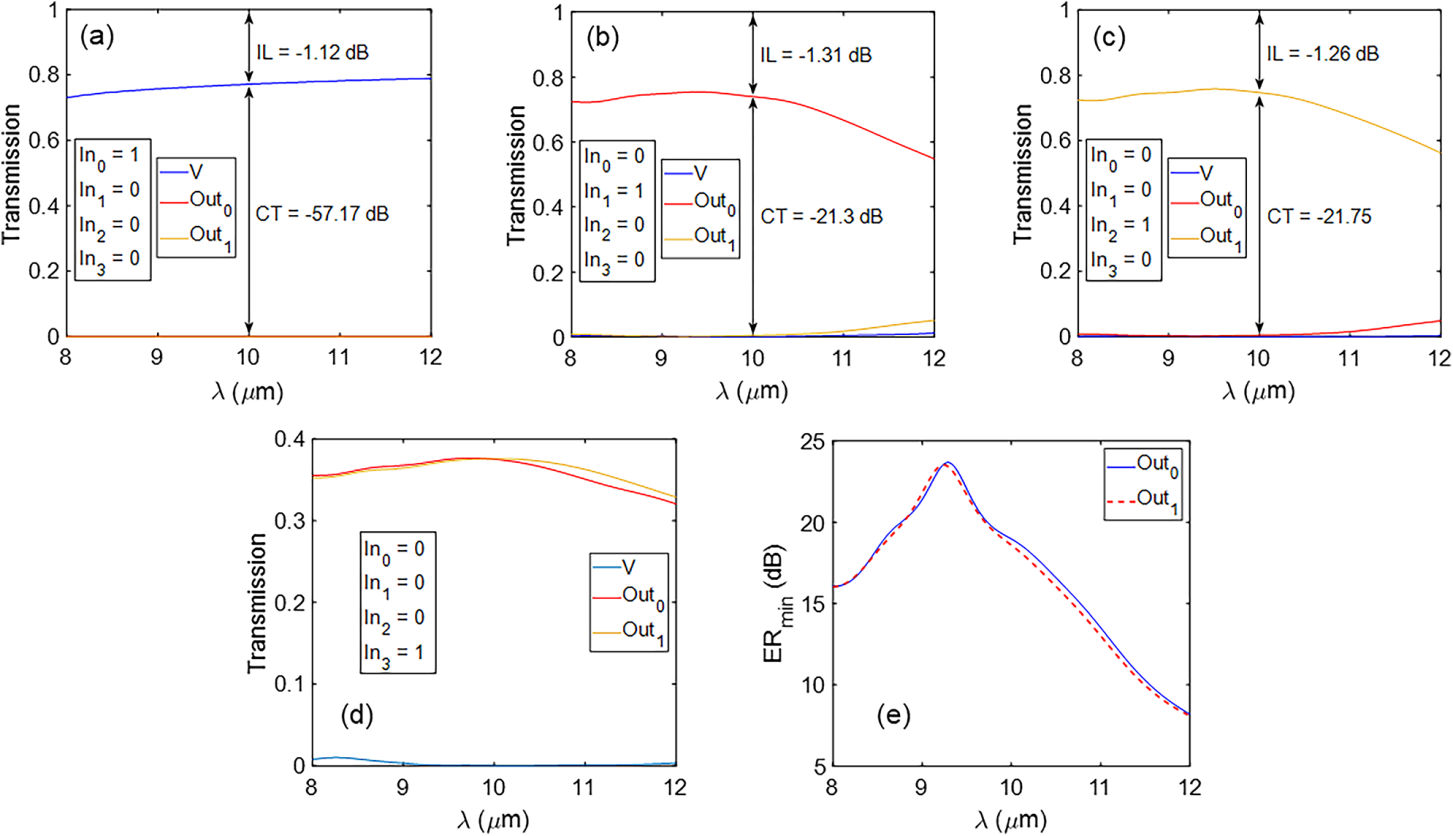
Transmission spectra at the output ports for different conditions of the proposed encoder (**a**) In_0_ = ON, (**b**) In_1_ = ON, (**c**) In_2_ = ON, and (**d**) In_3_ = ON. (**e**) The *ER*_*min*_ spectrum for Out_0_ and Out_1_ ports.

The distribution of |*E*| for different cases of the proposed encoder at λ = 10 *μ*m is plotted in (Fig. 10). For this purpose, a surface monitor is used parallel to the graphene sheet with a distance of 5 nm. In Fig. 10a, only In_0_ is ON, and the other inputs are OFF; therefore, output V is ON. When only In_1_ is ON, stimulated SPPs propagate toward the Out_0_ port, and therefore, output Out_0_ becomes ON, as seen in (Fig. 10b). When In_2_ is ON, SPPs travel toward the port Out_1_ and change its logical state to ON (Fig. 10c). Lastly, when In_3_ is ON, SPP waves pass almost equally from the upper and lower waveguides toward the ports Out_0_ and Out_1_, and therefore, these two outputs are in the ON state, as observed in (Fig. 10d). In Fig. 11a–d, the transmission spectra of output ports for the four cases of the encoder are plotted. The performance of the proposed encoder can be measured by defining the minimum extinction ratio (*ER*_*min*_) parameter as follows^46^:

9 $$ER_{\min } \left( {dB} \right) = 10\log \frac{{T_{ON,\min } }}{{T_{OFF,\max } }}$$

where *T*_*ON,min*_ refers to the minimum transmission value in the logical “1” state (ON), and *T*_*OFF,max*_ refers to the maximum transmission value in the logical “0” state (OFF). Ignoring the output V, the *ER*_*min*_ values are plotted for Out_0_ and Out_1_ ports in (Fig. 11e). Although the bent Y-combiners are identical, a negligible difference is seen in *ER*_*min*_ values for Out_0_ and Out_1_. This is attributed to the presence of the straight waveguide adjacent to the In_1_ waveguide, which leads to very weak coupling between the In_0_ and In_1_ waveguides and the structure asymmetry along the *y* direction. The *ER*_*min*_ at λ = 10 *μ*m is about 19 dB for both Out_0_ and Out_1_ ports. In addition, the insertion loss (*IL*) of the device can be calculated from the transmission spectra of Fig. 11 as follows^26^:

10 $$IL\left( {dB} \right) = 10\log \frac{{P_{Out} }}{{P_{In} }}$$

The difference between the unity transmission and the transmission at any port determines the *IL*. The suggested encoder can be considered a system with an individual straight waveguide and two Y-combiners. The straight waveguide shows the minimum *IL*, while the maximum value of *IL* of -1.31 dB at λ = 10 *μ*m corresponds to the Ou1 port. In the worst case, the *IL* increases to -2.6 dB at λ = 12 *μ*m. The cross-talk (*CT*) is the power coupled from an ON waveguide to the adjacent OFF waveguide. If waveguide 1 is in the ON state and waveguide 2 is in the OFF state, *CT* can be calculated using the following equation^26^:

11 $$CT\left( {dB} \right) = 10\log \frac{{P_{2,OFF} }}{{P_{1,ON} }}$$

where *P*_1*,ON*_ is the power in waveguide 1, and *P*_2*,OFF*_ is the power in waveguide 2. The *CT* values are presented in transmission spectra shown in (Fig. 11a–c). In the worst case, the *CT* decreases to − 10.71 dB at λ = 12 *μ*m.

**Fig. 12:**
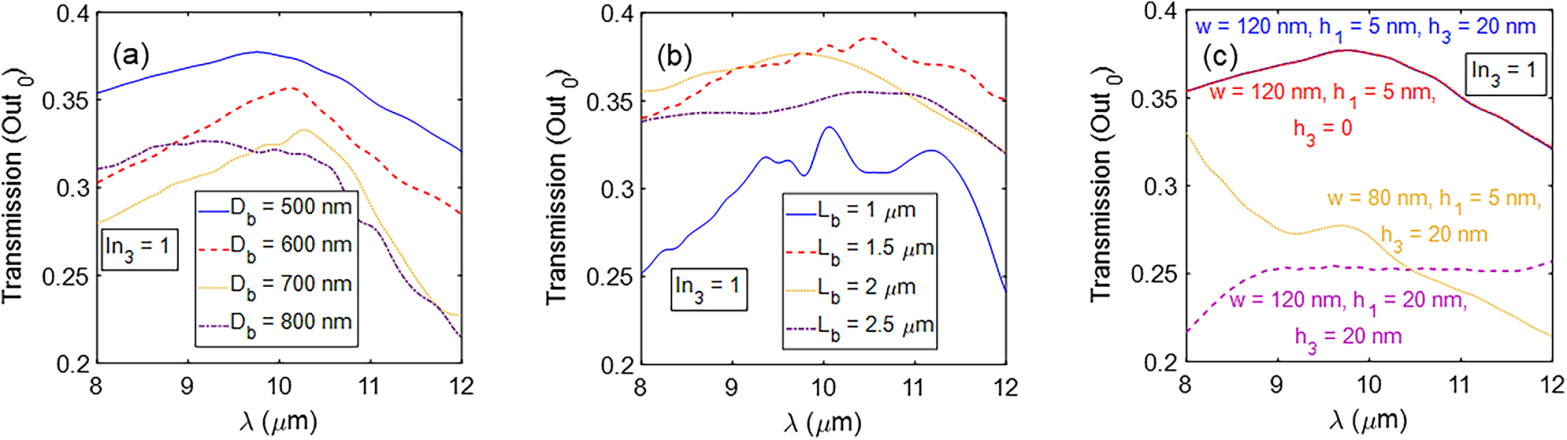
Transmission spectra at the Out_0_ port for various values of (**a**) *D*_*b*_, (**b**) *L*_*b*_, and (**c**) different geometric parameters of the encoder.

Using the two bent Y-combiners involves two geometric parameters, *D*_*b*_ and *L*_*b*_, and the impact of their change is investigated in the following. In Fig. 12a, the transmission spectrum of Out_0_ is depicted for different *D*_*b*_ values ranging from 500 to 800 nm with steps of 100 nm when In_3_ is ON. It is evident that higher *D*_*b*_ values lead to lower transmission values, especially in the longer wavelengths. It should be noted that lower *D*_*b*_ values than 500 nm increase the *CT*. The graphs in Fig. 12b do not show a linear relationship between the transmission spectra and the *L*_*b*_ parameter change. However, the transmission spectra change slightly for *L*_*b*_ values between 1.5 and 2.5 *μ*m, except for *L*_*b*_ = 1 *μ*m, where the device experiences high bending loss. In Fig. 12c, the transmission spectra at the Out_0_ port for different cases are compared when In_3_ is in the ON state. This figure proves the findings reported in the design of the basic waveguide. Moreover, it is seen that the existence of the spacer layer has no impact on the transmission spectrum of the encoder because it is far away from the stimulated fundamental mode region. The transmission spectra *h*_3_ = 20 nm and *h*_3_ = 0 are nearly identical. Finally, the results of the suggested encoder are compared with those of previously reported ones in (Table 1). It is observed that the proposed encoder has far better results than other previous works.

**Table 1:** Comparison between results of the proposed encoder and other previously reported encoders.

Ref	Waveguide	*ER*_*min*_ (dB)	*CT* (dB)	Footprint (μm^2^)
^47^	Photonic crystal	9.24	−11.76	792
^48^	Photonic crystal	16.33	−17.08	612
^49^	Photonic crystal	13.76	−16.53	1500
^50^	Photonic crystal	11.71	−15.01	723
^51^	Photonic crystal-Graphene	7.6	−10.46	127
^52^	Graphene plasmonic	14.44	−17.33	0.36
^27^	Graphene plasmonic	13.1	−16.22	1.92
This work	Graphene plasmonic	19	−21.3	4.25

## Conclusion

In summary, this paper presented an all-optical 4 × 2 encoder utilizing graphene-plasmonic waveguides. SPPs are stimulated in the gap between the graphene sheet and the Si strips. The effect of geometric parameters on the waveguide’s performance was analyzed through the 3D-FDTD method, demonstrating that introducing a gap between the graphene sheet and the Si strips significantly reduces losses. Additionally, the waveguiding characteristics can be controlled easily by applying an external voltage between the graphene sheet and the metal layer beneath the Si strips. The proposed encoder includes four input ports and three output ports to determine all the possible states of the input signals. The encoder demonstrates an *ER*_*min*_ of 19 dB at λ = 10 *μ*m while maintaining the *CT* and *IL* parameters at −21.3 and −1.31 dB, respectively. In the worst case, the *CT* and *IL* reach −10.71 and −2.6 dB at λ = 12 *μ*m, respectively. The device’s dimensions are 1.7 *μ*m × 2.5 *μ*m, offering an ultra-compact structure.

## Data Availability

The datasets used and/or analyzed during the current study are available from the corresponding author on reasonable request.
